# Tissue-specific expression of the SARS-CoV-2 receptor, angiotensin-converting enzyme 2, in mouse models of chronic kidney disease

**DOI:** 10.1038/s41598-021-96294-8

**Published:** 2021-08-19

**Authors:** Shunichiro Tsukamoto, Hiromichi Wakui, Kengo Azushima, Takahiro Yamaji, Shingo Urate, Toru Suzuki, Eriko Abe, Shohei Tanaka, Shinya Taguchi, Takayuki Yamada, Sho Kinguchi, Daisuke Kamimura, Akio Yamashita, Daisuke Sano, Masayuki Nakano, Tatsuo Hashimoto, Kouichi Tamura

**Affiliations:** 1 Department of Medical Science and Cardiorenal Medicine, Yokohama City University Graduate School of Medicine, 3-9 Fukuura, Kanazawa-ku, Yokohama, 236-0004 Japan; 2 Cardiovascular and Metabolic Disorders Program, Duke-NUS Medical School, Singapore, Singapore; 3 Department of Medicine, Mount Sinai Beth Israel, New York, NY USA; 4 Department of Molecular Biology, Yokohama City University Graduate School of Medicine, Yokohama, Japan; 5 Department of Otorhinolaryngology, Head and Neck Surgery, School of Medicine, Yokohama City University, Yokohama, Japan; 6 Internal Medicine, Kanagawa Dental University, Yokosuka, Japan

**Keywords:** Kidney, Kidney diseases, Chronic kidney disease, Hypertension

## Abstract

Elevated angiotensin-converting enzyme 2 (ACE2) expression in organs that are potential targets of severe acute respiratory syndrome coronavirus 2 may increase the risk of coronavirus disease 2019 (COVID-19) infection. Previous reports show that ACE2 alter its tissue-specific expression patterns under various pathological conditions, including renal diseases. Here, we examined changes in pulmonary ACE2 expression in two mouse chronic kidney disease (CKD) models: adenine-induced (adenine mice) and aristolochic acid-induced (AA mice). We also investigated changes in pulmonary ACE2 expression due to renin–angiotensin system (RAS) blocker (olmesartan) treatment in these mice. Adenine mice showed significant renal functional decline and elevated blood pressure, compared with controls. AA mice also showed significant renal functional decline, compared with vehicles; blood pressure did not differ between groups. Renal ACE2 expression was significantly reduced in adenine mice and AA mice; pulmonary expression was unaffected. Olmesartan attenuated urinary albumin excretion in adenine mice, but did not affect renal or pulmonary ACE2 expression levels. The results suggest that the risk of COVID-19 infection may not be elevated in patients with CKD because of their stable pulmonary ACE2 expression. Moreover, RAS blockers can be used safely in treatment of COVID-19 patients with CKD.

## Introduction

Since January 2020, coronavirus disease 2019 (COVID-19), caused by severe acute respiratory syndrome coronavirus 2 (SARS-CoV-2), has been a global public health problem. SARS-CoV-2 infection is established when the viral S protein binds to host angiotensin-converting enzyme 2 (ACE2) and enters the cell^1,2^. Therefore, elevated ACE2 expression in organs that are potential targets of SARS-CoV-2 may increase the risk of COVID-19 infection.

ACE2 is an enzyme that plays an important role in the renin–angiotensin system (RAS), where it converts angiotensin (Ang) II to Ang1-7 and forms a component of the ACE2-Ang1-7-MAS receptor axis^3^. It also exerts an organ-protective effect by counteracting the activity of the ACE-Ang II-angiotensin type 1 (AT1) receptor axis^4–7^. Previous reports have shown that ACE2 alters its tissue-specific expression patterns under various conditions, including cardiovascular diseases, diabetes mellitus (DM), and renal diseases^8–11^. Pulmonary ACE2 expression, which is particularly important for SARS-CoV-2 infection, is reduced in lipopolysaccharide-induced acute respiratory distress syndrome^12^. Another report shows that ACE2 expression is higher in older men^13^. In addition, nasal and branchial ACE2 expression is higher in adults than in children^14^. However, to the best of our knowledge, few studies have examined how pulmonary ACE2 expression is altered in other diseases.

Patients with chronic kidney disease (CKD) have a risk of severe COVID-19 disease, but there is no evidence of increased prevalence of COVID-19 in CKD patients^15–21^. Considering the fact that ACE2 is required for COVID-19 infection, these findings may suggest that pulmonary ACE2 expression is not enhanced in patients with CKD. Notably, cellular ACE2 expression is presumably associated with susceptibility to the risk of infection by SARS-CoV, which is related to SARS-CoV-2^22^. The fact that children with low ACE2 expression are less susceptible to COVID-19 than adults^23^ also supports these hypotheses. Therefore, we conducted experiments to examine changes in pulmonary ACE2 expression in two types of CKD model mice: adenine-induced (i.e., adenine mice) and aristolochic acid (AA)-induced (i.e., AA mice) to address this hypothesis.

The relationship of ACE2 expression with RAS blockers has also been investigated. Thus far, the use of RAS blockers has been shown to upregulate organ ACE2 expression in some instances^24–27^. Recent epidemiological investigations have shown that the use of RAS blockers does not increase the risk of COVID-19^28–30^. Furthermore, ACE2 expression in the lungs was not enhanced when RAS blockers were administered to normal mice^31^. However, there have been no reports concerning the effects of RAS blockers on pulmonary ACE2 expression in the context of CKD; this point is essential for future management of COVID-19 and its sequelae. Here, we investigated changes in pulmonary ACE2 expression due to RAS blocker treatment in CKD model mice.

## Results

### Adenine-induced CKD model mice showed significant weight loss, renal functional decline, and elevated blood pressure (BP), compared with controls

**Figure 1:**
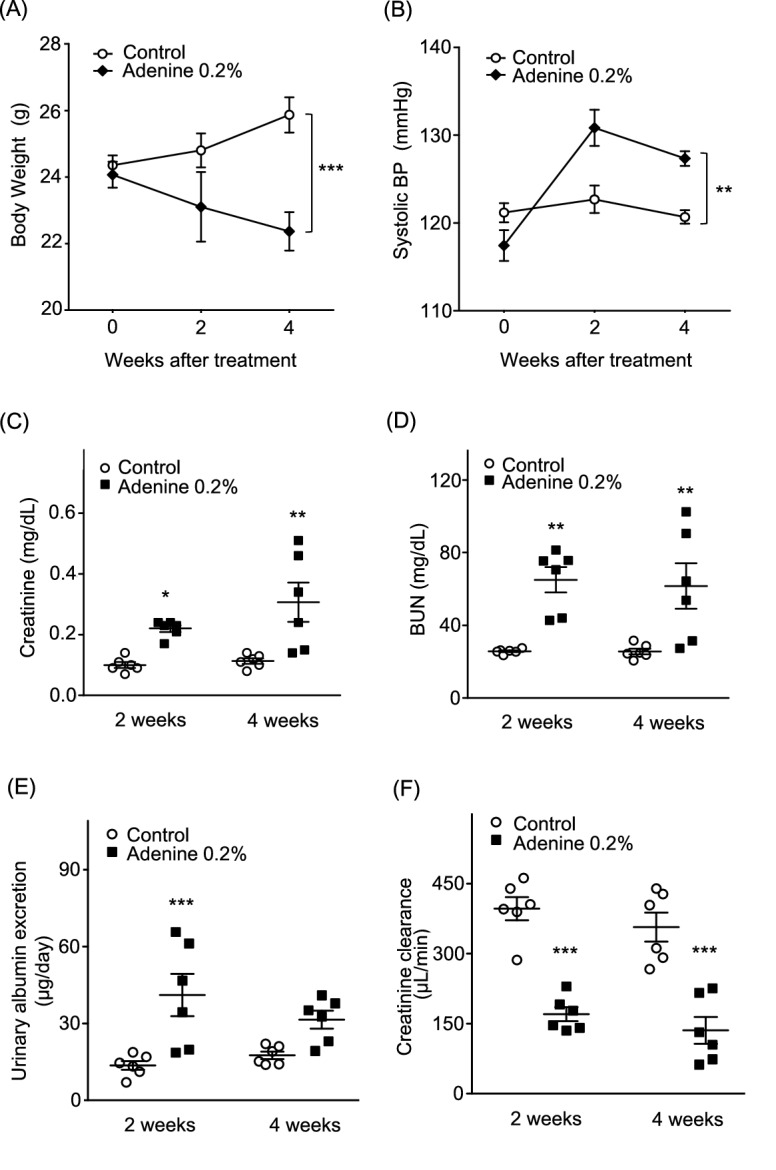
Body weight, blood pressure, and renal functions in adenine-induced CKD model mice. (**A**) Body weight changes in control and adenine groups. (**B**) Systolic blood pressure in control and adenine groups at 0, 2, or 4 weeks after treatment. (**C**) Plasma creatinine level, (**D**) BUN level, (**E**) urinary albumin excretion, and (**F**) creatinine clearance were measured at 2 or 4 weeks after treatment in both control and adenine groups. Values are expressed as mean ± standard error of the mean (six mice per group). *P* values, versus control: *P < 0.05; **P < 0.01; ***P < 0.001. CKD, chronic kidney disease; BP, blood pressure; BUN, blood urea nitrogen.

Baseline body weight (BW) and systolic BP were identical between control and adenine groups. BW increased over time in the control group, while it decreased in the adenine group; this BW gain was significantly different between groups (Fig. 1A). Systolic BP was significantly elevated in the adenine group, compared with the control group, over time (Fig. 1B). At 2 weeks, urinary albumin excretion was significantly elevated in the adenine group, compared with the control group (adenine mice 41.0 ± 8.3 μg/day vs. control 13.6 ± 1.7 μg/day, *P* < 0.001; Fig. 1E). There were also significant enhancements of plasma creatinine and blood urea nitrogen (BUN) levels in the adenine group, compared with the control group at 2 or 4 weeks (creatinine: 2 weeks: adenine mice 0.22 ± 0.01 mg/dL vs. control 0.10 ± 0.01 mg/dL, *P* < 0.05; 4 weeks: adenine mice 0.31 ± 0.06 mg/dL vs. control 0.11 ± 0.01 mg/dL, *P* < 0.01; BUN: 2 weeks: adenine mice 65.0 ± 7.0 mg/dL vs. control 25.7 ± 0.5 mg/dL, *P* < 0.01; 4 weeks: adenine mice 61.7 ± 12.5 mg/dL vs. control 25.6 ± 1.6 mg/dL, *P* < 0.01; Fig. 1C, D). Moreover creatinine clearance also showed a significant reduction in the adenine group (2 weeks: adenine mice 170 ± 15 μL/min vs. control 397 ± 25 μL/min, *P* < 0.001; 4 weeks: adenine mice 136 ± 29 μL/min vs. control 357 ± 31 μL/min, *P* < 0.001; Fig. 1F).

### ACE2 expression in the kidneys was significantly reduced in adenine-induced CKD model mice, compared with controls, while ACE2 expression in the lungs did not differ between groups

**Figure 2:**
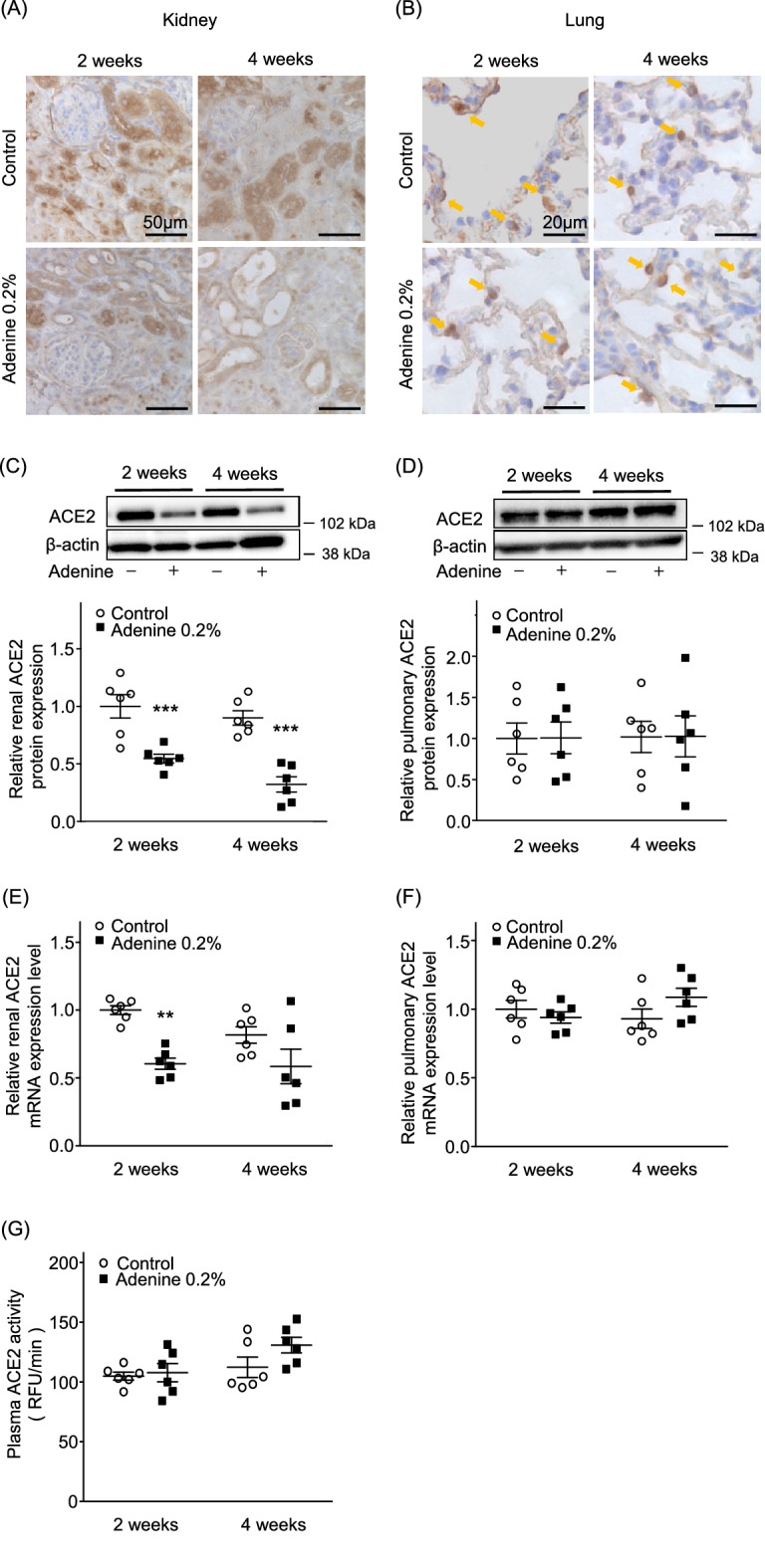
Renal and pulmonary ACE2 expression levels and plasma ACE2 activity in adenine-induced CKD mice. (**A**) Representative image of renal ACE2 immunostaining at 2 or 4 weeks after treatment in adenine and control groups. Bars = 50 µm. (**B**) Representative image of pulmonary ACE2 immunostaining at 2 or 4 weeks after treatment in adenine and control groups. Bars = 20 µm. (**C**) Renal ACE2 protein expression levels and (**E**) mRNA expression levels in adenine and control groups. (**D**) Pulmonary ACE2 protein expression levels and (**F**) mRNA expression levels in adenine and control groups. (**G**) Plasma ACE2 activity in adenine and control groups. Values are expressed as mean ± standard error of the mean (six mice per group). *P* values, versus control: *P < 0.05; **P < 0.01; ***P < 0.001.

Renal histology showed heterogeneous enlargement and atrophy of the tubules, as well as cellular infiltration into the renal interstitium, in adenine mice. ACE2 staining was observed mainly in the proximal tubules in both groups, but the degree of staining was reduced in the adenine group (Fig. 2A). ACE2 protein levels in the kidneys (estimated by western blotting analysis) showed a significant reduction in the adenine group, compared with the control group, at both 2 and 4 weeks (2 weeks: adenine mice 0.55 ± 0.04 vs. control 1.00 ± 0.10, *P* < 0.001; 4 weeks: adenine mice 0.32 ± 0.07 vs. control 0.90 ± 0.07, *P* < 0.001; Fig. 2C). In addition, renal ACE2 mRNA expression showed a significant reduction in the adenine group at 2 weeks (adenine mice 0.61 ± 0.04 vs. control 1.00 ± 0.03, *P* < 0.001; Fig. 2E); a similar tendency for reduction was observed at 4 weeks, although the difference was not statistically significant (Fig. 2E). Lung histology findings did not differ between groups; ACE2 staining was observed in type 2 alveolar epithelial cells (Fig. 2B). There were no differences in ACE2 protein or mRNA expression levels in the lungs at 2 or 4 weeks (Fig. 2D, F). The difference in plasma ACE2 activity was not statistically significant (Fig. 2G).

### AA-induced CKD model mice showed significant weight loss and renal functional decline, compared with the vehicle group, while BP did not differ between groups

**Figure 3:**
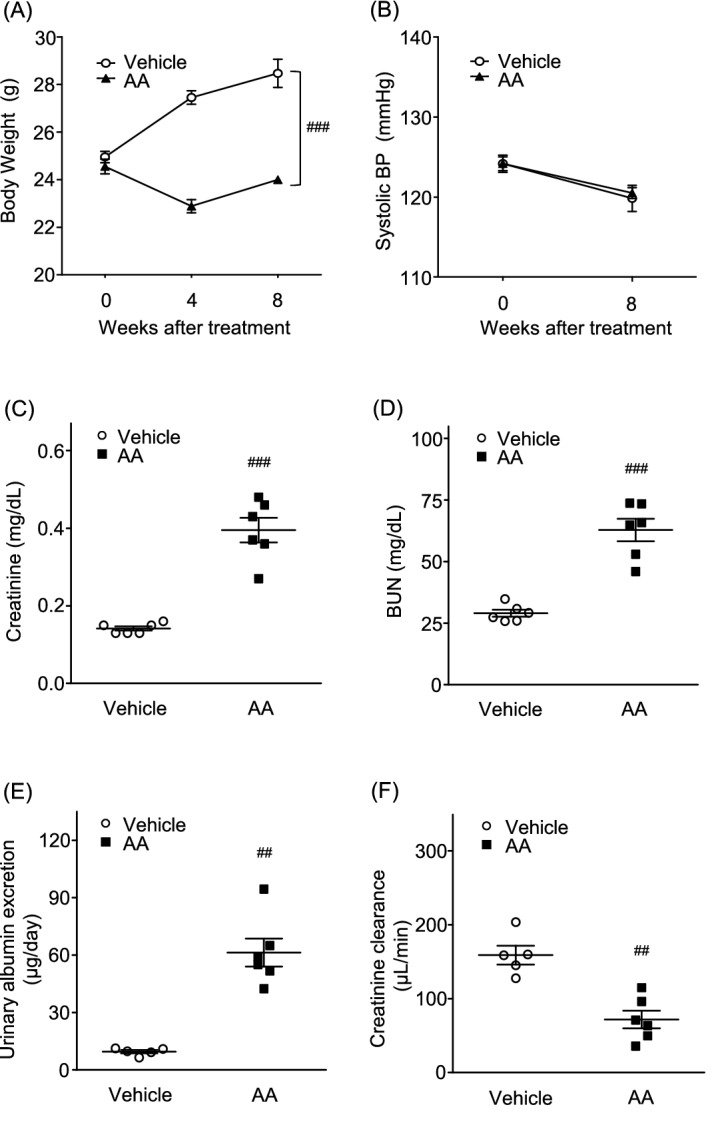
Body weight, blood pressure, and renal functions in aristolochic acid (AA)-induced CKD model mice. (**A**) Body weight changes in vehicle and AA groups. (**B**) Systolic blood pressure in vehicle and AA groups at 0, 4, or 8 weeks after treatment. (**C**) Plasma creatinine level, (**D**) BUN level, (**E**) urinary albumin excretion, and (**F**) creatinine clearance were measured at 8 weeks after treatment in vehicle and AA groups. Values are expressed as mean ± standard error of the mean (5–6 mice per group). P values, versus vehicle: ^#^*P* < 0.05; ^##^*P* < 0.01; ^###^*P* < 0.001. CKD, chronic kidney disease; BP, blood pressure; BUN, blood urea nitrogen.

Baseline BW and systolic BP were identical between vehicle and AA groups. BW increased over time in the vehicle group, while it decreased in the AA group; this BW gain was significantly different between groups (Fig. 3A). However, there was no difference in systolic BP between groups (Fig. 3B). Compared with the vehicle group, there were significant enhancements of plasma creatinine and BUN levels, as well as urinary albumin excretion, in the AA group (creatinine: AA mice 0.40 ± 0.03 mg/dL vs. vehicle 0.14 ± 0.01 mg/dL, *P* < 0.001; BUN: AA mice 62.8 ± 4.6 mg/dL vs. vehicle 29.0 ± 1.4 mg/dL, *P* < 0.001; urinary albumin excretion: AA mice 61.3 ± 7.3 μg/day vs. vehicle 9.5 ± 0.9 μg/day, *P* < 0.001; Fig. 3C–E). Creatinine clearance also showed a significant reduction in the AA group (AA mice 72 ± 12 μL/min vs. vehicle 159 ± 13 μL/min, *P* < 0.001; Fig. 3F).

### ACE2 expression in the kidneys was significantly reduced in AA-induced CKD model mice, compared with controls, while pulmonary ACE2 protein levels did not differ between groups

**Figure 4:**
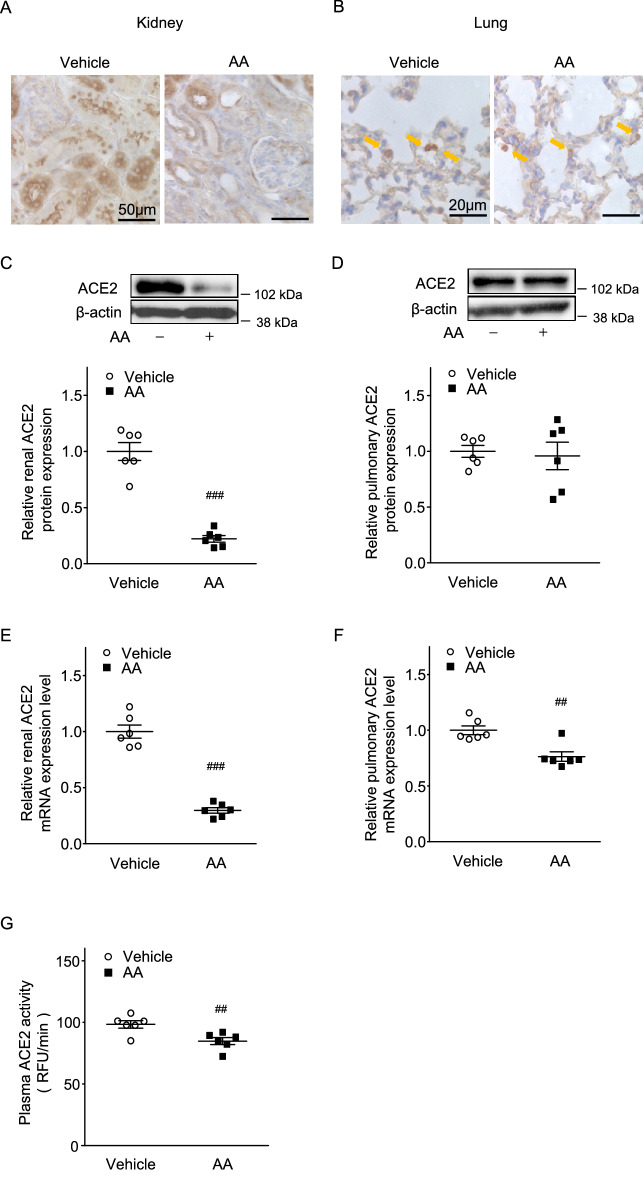
Renal and pulmonary ACE2 expression levels and plasma ACE2 activity in aristolochic acid (AA)-induced CKD model mice. (**A**) Representative image of renal ACE2 immunostaining at 8 weeks after treatment in vehicle and AA groups. Bars = 50 µm. (**B**) Representative image of pulmonary ACE2 immunostaining at 8 weeks after treatment in vehicle and AA groups. Bars = 20 µm. (**C**) Renal ACE2 protein expression levels and (**E**) mRNA expression levels in vehicle and AA groups. (**D**) Pulmonary ACE2 protein expression levels and (**F**) mRNA expression levels in vehicle and AA groups. (**G**) Plasma ACE2 activity in vehicle and AA groups. Values are expressed as mean ± standard error of the mean (six mice per group). *P* values, versus vehicle: ^#^*P* < 0.05; ^##^*P* < 0.01; ^###^*P* < 0.001. CKD, chronic kidney disease.

Renal histology analysis showed tubular atrophy and cellular infiltration into the renal interstitium in the AA group. ACE2 staining was observed mainly in the proximal tubules in both groups, but the degree of staining was reduced in the AA group (Fig. 4A). ACE2 protein levels in the kidneys showed a significant reduction in the AA group, compared with the vehicle group (AA mice 0.22 ± 0.03 vs. vehicle 1.00 ± 0.08, *P* < 0.001; Fig. 4C). In addition, renal ACE2 mRNA expression was significantly reduced in the AA group (AA mice 0.30 ± 0.02 vs. vehicle 1.00 ± 0.06, *P* < 0.001; Fig. 4E). Lung histology findings did not differ between groups; ACE2 staining was observed in type 2 alveolar epithelial cells (Fig. 4B) and there was no difference in pulmonary ACE2 protein level between groups (Fig. 4D). ACE2 mRNA expression in the lungs was significantly lower in the AA group than in the vehicle group (AA mice 0.76 ± 0.04 vs. vehicle 1.00 ± 0.04, *P* < 0.01; Fig. 4F). Moreover, there was a significant reduction in plasma ACE2 activity in the AA group (AA mice 84.8 ± 2.6 RFU/min vs. vehicle 98.3 ± 3.0 RFU/min, *P* < 0.01; Fig. 4G).

### Olmesartan attenuated enhancements of BP and urinary albumin excretion and reduction of weight loss in adenine-induced CKD model mice

**Figure 5:**
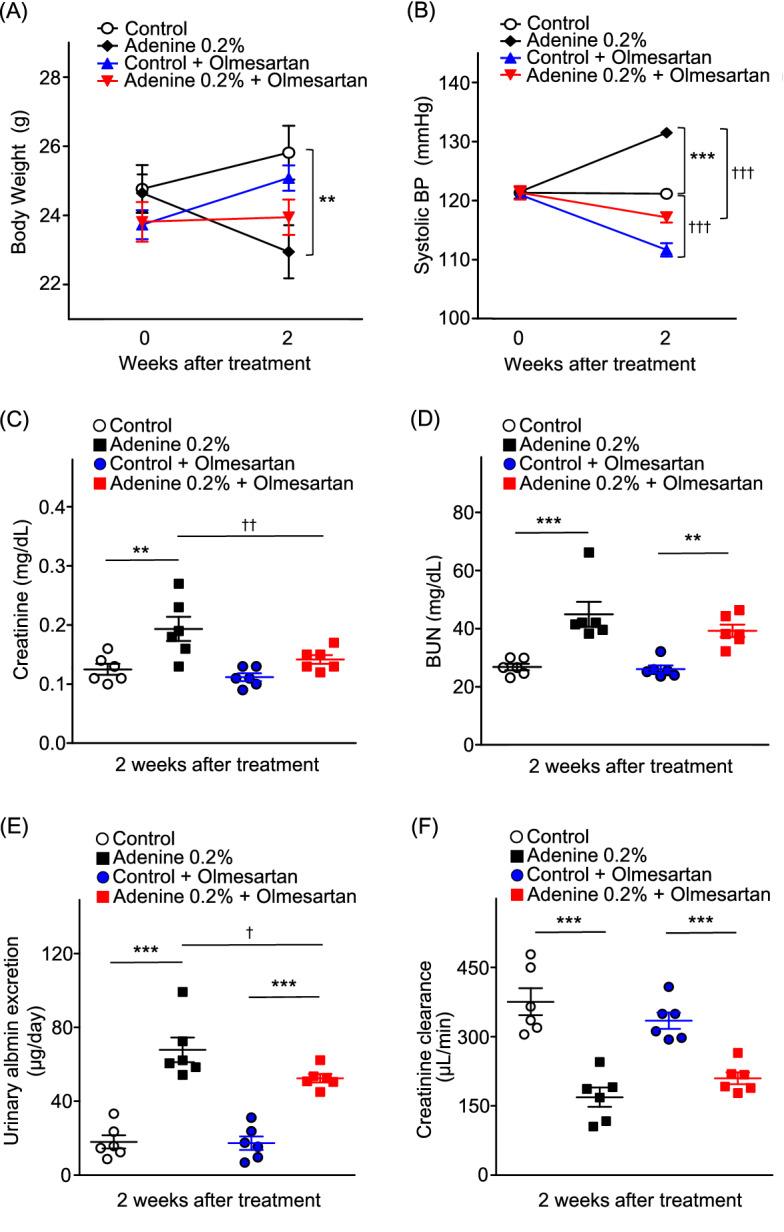
Effects of olmesartan administration on body weight, blood pressure, and renal function. (**A**) Body weight and (**B**) blood pressure in control, adenine, control-olmesartan, and adenine-olmesartan groups. (**C**) Plasma creatinine level, (**D**) plasma BUN level, (**E**) urinary albumin excretion, and (**F**) creatinine clearance in control, adenine, control-olmesartan, and adenine-olmesartan groups. Values are expressed as mean ± standard error of the mean (six mice per group). *P* values, versus control: *P < 0.05; **P < 0.01; ***P < 0.001; versus without olmesartan: ^†^*P* < 0.05; ^††^*P* < 0.01; ^†††^*P* < 0.001. BP, blood pressure; BUN, blood urea nitrogen.

Olmesartan treatment attenuated weight loss in adenine mice (Fig. 5A). Olmesartan treatment also significantly reduced systolic BP in both control and adenine groups (adenine mice 131.5 ± 0.4 mmHg vs. control 121.2 ± 0.7 mmHg, *P* < 0.001; olmesartan mice 111.7 ± 1.1 mmHg vs. control 121.2 ± 0.7 mmHg, *P* < 0.001; adenine + olmesartan mice 117.2 ± 0.9 mmHg vs. adenine mice 131.5 ± 0.4 mmHg, *P* < 0.001; Fig. 5B).

In adenine mice, olmesartan treatment improved plasma creatinine level (adenine mice 0.19 ± 0.02 vs. control 0.13 ± 0.01, *P* < 0.01; adenine + olmesartan mice 0.14 ± 0.01 vs. adenine mice 0.19 ± 0.02, *P* < 0.01; Fig. 5C) and suppressed urinary albumin excretion (adenine mice 67.9 ± 6.7 μg/day vs. control 18.0 ± 3.6 μg/day, *P* < 0.001; adenine + olmesartan mice 52.5 ± 2.3 vs. olmesartan mice 17.4 ± 3.7 μg/day, *P* < 0.001; adenine-olmesartan mice 52.5 ± 2.3 μg/day vs. adenine mice 67.9 ± 6.7 μg/day, *P* < 0.05; Fig. 5E).

### Olmesartan did not affect ACE2 expression

**Figure 6:**
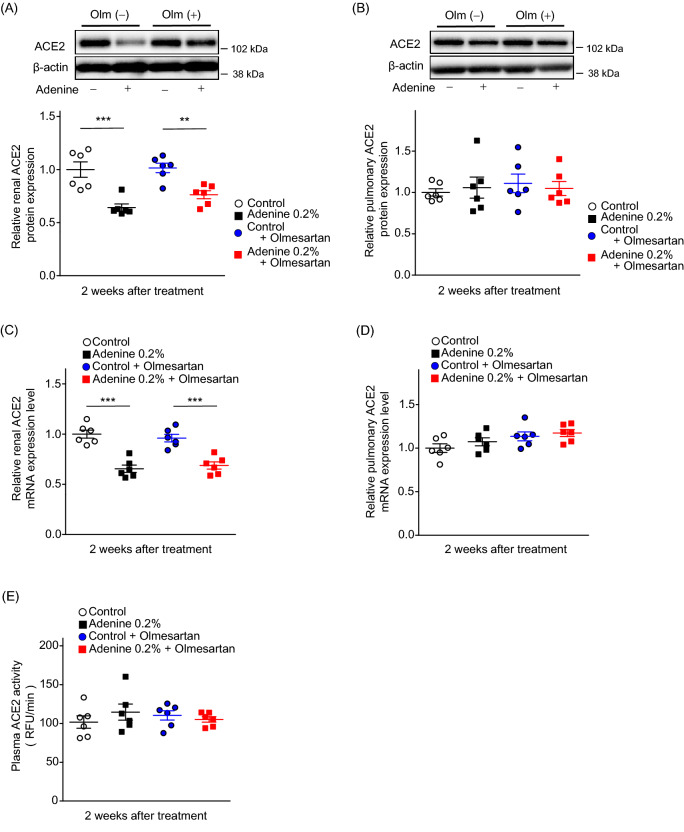
Effects of olmesartan administration on renal and lung ACE2 expression levels and plasma ACE2 activity. (**A**) Renal ACE2 protein expression levels and (**C**) mRNA expression levels in control, adenine, control-olmesartan, and adenine-olmesartan groups. (**B**) Pulmonary ACE2 protein expression levels and (**D**) mRNA expression levels in control, adenine, control-olmesartan, and adenine-olmesartan groups. (**E**) Plasma ACE2 activity in control, adenine, control-olmesartan, and adenine-olmesartan groups. Values are expressed as mean ± standard error of the mean (six mice per group). *P* values, versus control: *P < 0.05; **P < 0.01; ***P < 0.001. Olm, olmesartan.

In the control and adenine group, neither mRNA nor protein expression levels of ACE2 in the kidney were altered by olmesartan treatment (Fig. 6A, C). There were no changes in pulmonary ACE2 mRNA or protein expression levels between the control and adenine groups (Fig. 6B, D). There was also no significant difference in plasma ACE2 activity (Fig. 6E). ACE2 mRNA expression in the upper respiratory tract (pharynx) was also examined, but no significant difference was observed between the groups (Supplementary Figure S1).

## Discussion

The COVID-19 pandemic has had a profound impact worldwide. For elderly individuals and people with comorbidities, information concerning the infection risk and severity of COVID-19 is a critical consideration. There have been numerous epidemiological studies of COVID-19 worldwide, which have shown that patients with CKD are more likely to develop severe COVID-19^17^ but the morbidity of COVID-19 may not be elevated in patients with CKD^15–21^. Notably, the results of several meta-analysis revealed that the prevalence of CKD was 1.7–5.2% in patients with COVID-19^15–17,21^. In addition, an analysis of 5,700 patients with COVID-19 in New York area showed that 8.5% had underlying renal disease^19^. Given that the estimated prevalence of CKD is 9.1% worldwide^18^ and approximately 15% in the United States^20^, the risk of SARS-CoV-2 infection may not increase in relation to the presence of CKD.

ACE2 plays an important role in the transmission of COVID-19^1,2^. In addition, the level of ACE2 expression in airway cells is associated with the risk of infection by SARS-CoV, a similar coronavirus to the causative agent of COVID-19^22^. Because COVID-19 is a respiratory disease, ACE2 expression in the lungs is an important component of disease transmission. In the present study, we hypothesized that the lack of increase in pulmonary ACE2 expression in CKD is linked to the absence of elevated COVID-19 morbidity. This hypothesis is supported by the fact that children with low ACE2 expression are less susceptible to COVID-19 than adults.

We used two types of CKD model mice in our experiments to examine changes in pulmonary ACE2 expression in connection with CKD pathogenesis. Adenine-induced CKD is a representative animal model of disease^32^. Excessive adenine administration can induce CKD by causing renal tubular obstruction and degeneration, as well as renal interstitial fibrosis, due to the deposition of 2–8 dihydroxyadenine^32,33^. In contrast, AA-induced CKD is caused by inducing fibrosis from AA-induced tubular damage^34^. These two models were chosen because BP has been shown to increase in the adenine-induced CKD model^35^, while it does not increase in the AA-induced CKD model^36^. Notably, hypertension is a common complication observed in patients with CKD; we presumed that it would be useful to investigate whether the presence or absence of hypertension influences pulmonary ACE2 expression in CKD pathogenesis. The results showed that the renal ACE2 protein levels were reduced in both CKD models. In adenine mice, there was no significant difference in mRNA levels at 4 weeks, but there was a decrease in protein levels. One of the reasons for this discrepancy may be the involvement of translational repression. Lambert DW, et al. showed that miR-421 repressed the translation of ACE2 protein without affecting ACE2 transcript levels^37^. Another study also showed that serum miR-421 was increased in CKD or hypertension patients^38,39^. Thus, in the adenine mice group with a longer period of CKD and hypertension (4 weeks), miR-421 might be increased and suppressed protein translation, resulting in a discrepancy between mRNA and protein expression. Pulmonary ACE2 protein levels did not differ in both CKD models compared to the controls and vehicles. Although it is intriguing that pulmonary ACE2 mRNA expression was reduced in the AA-induced CKD model, our findings were more notable because we found no difference in pulmonary ACE2 protein levels, which directly influence SARS-CoV-2 infection.

In addition, plasma ACE2 activity did not differ in both CKD models compared to the controls and vehicles. Soluble ACE2 in plasma is considered to be cleaved and released from tissue cells. Increased plasma ACE2 activity has been reported to be associated with increase in release of ACE2 protein from tissues, suggesting a decrease in ACE2 protein in the tissues^40^. Thus, the results of the present study that plasma ACE2 activity was not changed in the CKD state indicates that the release of ACE2 protein expressed in tissues is not changed in the CKD state, which supports the findings that ACE2 protein expression in tissues is unchanged in the context of CKD.

The relationship between COVID-19 and RAS blockers is an ongoing focus of research discussions. Previous animal studies showed that RAS blockers upregulate tissue ACE2 expression, which led to fears that the use of RAS blockers may increase the risk of SARS-CoV-2 infection. However, the use of RAS blockers in those prior studies only led to upregulation of tissue ACE2 under specific pathological conditions^24,27^; this upregulation did not exceed the normal range. Another study examining pulmonary and renal ACE2 expression in normal mice treated with RAS blockers showed no increase, compared with the control group^31^. On the other hand, there was a report that RAS blockers such as candesartan and captopril increased pulmonary ACE2 expression in normal rats^41^. In real-world clinical practice, some epidemiological studies concerning the use of RAS blockers in patients with COVID-19 have demonstrated that the use of those drugs does not increase the risk of COVID-19^28–30^. However, the effect of RAS blockers on pulmonary ACE2 expression is still controversial. No previous studies have examined the effects of RAS blockers on pulmonary ACE2 expression in the context of CKD, although RAS blockers are essential for the treatment of patients with CKD. The present study was performed to address this gap in the literature. To more closely model real-world clinical practice, we used the adenine-induced CKD model, which exhibits concurrent hypertension. The results showed that administration of olmesartan, an RAS blocker, did not enhance pulmonary ACE2 expression, compared with controls. In addition, ACE2 mRNA expression in the upper respiratory tract, the initial target of SARS-CoV-2 infection, was not enhanced in response to olmesartan treatment. Renal ACE2 protein levels were reduced in the CKD model; administration of olmesartan did not increase renal ACE2 protein levels in the control and adenine mice. The lack of restoration in renal ACE2 in olmesartan-treated adenine mice might have been due to the use of a different CKD model, compared with a previous study^27^, as well as the relatively short duration of angiotensin receptor blocker treatment^25,27^. However, olmesartan treatment improved several renal failure parameters, including urinary albumin excretion. These results support the continued use of RAS blockers in patients with COVID-19 who have CKD.

This study had some limitations. First, it only examined changes in pulmonary ACE2 expression in CKD model mice, whereas it did not directly investigate SARS-CoV-2 infection. Second, we observed the adenine mice for 4 weeks and the AA mice for 8 weeks in this study, but the results may be different in a longer period of observation. Third, ACE2 expression in the lungs may differ between mice and humans. Fourth, in patients with CKD, COVID-19 severity is likely to be greater, but we could not refer to the underlying mechanism of severity. Fifth, it is not only the expression level of pulmonary ACE2 that determines the susceptibility to COVID-19, but it may also be affected by many other factors.

In conclusion, we found no change in pulmonary ACE2 protein expression in CKD model mice, regardless of hypertension status. In addition, the use of angiotensin receptor blocker treatment in CKD model mice did not increase pulmonary ACE2 expression. These results suggest the hypothesis that the risk of COVID-19 morbidity may not be elevated in patients with CKD because of their stable pulmonary ACE2 expression. The findings also provide important basic scientific evidence that RAS blockers can be used safely in treatment of patients with COVID-19 who have CKD.

## Material and methods

### Animals

This study was performed in accordance with the National Institutes of Health guidelines for the use of experimental animals. All animal experiments were reviewed and approved by the Animal Studies Committee of Yokohama City University (Approval Number: FA20-027), which were in compliance with the ARRIVE guidelines. Efforts were made to minimize the number of animals used and ensure minimal sufferings. The mice were housed in a controlled environment with a 12-h light–dark cycle at a temperature of 25 °C. The mice were allowed free access to food and water.

### CKD model mice

The experiments were performed using 8–9-week-old male C57BL/6 J mice, following 1-week acclimatization in all groups. In the adenine experiment, mice were fed a diet mixed with 0.2% adenine (0.2% adenine + 0.3% NaCl, 3.6 kcal/g, CE-2; CLEA, Tokyo, Japan; adenine group) or a standard diet (0.3% NaCl, 3.6 kcal/g, CE-2; CLEA; control group) for 2 or 4 weeks. In the AA experiment, mice were intraperitoneally injected with AA (3 mg/kg) twice per week for 4 weeks, followed by a 4-week recovery period; the vehicle group was injected with vehicle (75% dimethyl sulfoxide).

In the olmesartan treatment experiment, mice were randomly divided into four experimental groups: (1) control group; (2) adenine group, fed a diet mixed with 0.2% adenine; (3) control-olmesartan group, treated with the AT1 receptor antagonist olmesartan in drinking water (4 mg/kg/day; Daiichi Sankyo Chemical Pharma Co., Ltd, Tokyo, Japan); and (4) adenine-olmesartan group, which doses identical to those in groups 2 and 3.

### BP measurement

Systolic BP was measured by the tail-cuff method (BP-Monitor MK-2000; Muromachi Kikai Co., Tokyo, Japan), as described previously^42,43^. All measurements were performed between 9:00 and 14:00 h. At least 10 measurements were performed in each mouse and the mean value was used for analysis.

### Real-time quantitative reverse transcription polymerase chain reaction analysis

Total RNA was extracted from lung, renal and pharynx tissue using ISOGEN (Nippon Gene, Tokyo, Japan); cDNA was synthesized using the SuperScript III First-Strand System (Invitrogen, Carlsbad, CA, USA). Real-time quantitative reverse transcription polymerase chain reaction analysis was performed using an ABI PRISM 7000 Sequence Detection System; reverse transcription products were incubated with TaqMan PCR Master Mix and a custom TaqMan probe (Applied Biosystems, Foster City, CA, USA), as described previously^44,45^. The following TaqMan probe was used: ACE2 (Mn01159003_m1). mRNA levels were normalized to those of 18S rRNA.

### Western blotting analysis

Protein expression was analyzed by western blotting using tissue homogenates, as described previously^45,46^. Briefly, total protein extract was prepared from tissues with sodium dodecyl sulfate-containing sample buffer. The protein concentration of each sample was measured with NanoDrop One (Thermo Fisher Scientific), using bovine serum albumin as the standard. Equal amounts of protein extract from each tissue samples (lung tissue: 24 μg, kidney tissue: 10 μg) were fractionated on a 5–20% polyacrylamide gel (Atto, Tokyo, Japan). The separated proteins were then transferred to a polyvinylidene difluoride membrane using the iBlot Dry Blotting System (Invitrogen). Membranes were blocked for 1 h at room temperature with phosphate-buffered saline containing 5% skim milk powder. Membranes were incubated with primary antibodies for ACE2 (Ab108252 1:500 [lung] or 1:1000 [kidney], Abcam, Cambridge, MA, USA) and β-actin (A5441 1:10,000, Sigma-Aldrich, St. Louis, MO, USA). Membranes were washed and then incubated with secondary antibodies for 60 min at room temperature. The sites of antibody–antigen reactions were visualized by enhanced chemiluminescence substrate (Merck, Kenilworth, NJ, USA). Images were analyzed quantitatively using a ChemiDoc Touch (Bio Rad, Hercules, CA, USA). To compare ACE2 protein expression levels in lung and renal tissues in normal mice, additional western blotting was performed on the same membrane (Supplementary Figure S2). The amount of protein described was fractionated into gels ((A) lung: 24/10/1 μg, kidney: 24/10/1 μg, (B) lung: 24/12 μg, kidney: 0.2/0.15/0.1 μg); the same primary antibody for ACE2 (Ab108252 1:1000, Abcam, Cambridge, MA, USA) was used. To examine the specificity of the antibody, an ACE2-selective blocking peptide (ab198988) was used. Western blotting showed a single protein band, which was abolished by an ACE2-selective blocking peptide (Supplementary Figure S3).

### Immunohistochemical analysis

Lung and renal tissues from mice were fixed with 4% paraformaldehyde and subsequently embedded in paraffin. Four-micrometer-thick sections were dewaxed and rehydrated; antigen retrieval was performed by microwave heating. The sections were blocked to reduce endogenous biotin activity using peroxidase blocking reagent (Dako, Carpinteria, CA, USA) and treated for 60 min with 10% normal goat serum in phosphate-buffered saline. The sections were then incubated with anti-ACE2 antibody diluted to 1:500 (Ab15348, Abcam). To examine the specificity of the antibody, immunostaining was also examined by omission of primary Ab and using an ACE2-selective blocking peptide (ab15352). ACE2 staining in lung and renal tissues was observed, which was not observed when the antibody was preabsorbed with an ACE2-selective blocking peptide (ab15352) or omission of ACE2 antibody. (Supplementary Figure S4).

### Biochemical analysis

After inhaling 5% isoflurane anaesthesia, Blood samples were collected by cardiac puncture in the fed state by heart punctures. Experimental animals were killed humanely after anaesthesia. Whole blood samples were centrifuged at 3000 rpm (MR-150, Tomy Seiko Co., Ltd., Tokyo, Japan) at 4 °C for 10 min to separate the plasma. The resulting plasma samples were stored at − 80 °C until use. Plasma creatinine, BUN, urinary creatinine, and urinary albumin levels were measured using a Hitachi 7180 autoanalyzer (Hitachi, Tokyo, Japan).

### Plasma ACE2 activity

ACE2 activity was measured by TechnoPro R&D Company (Tokyo, Japan) using the ACE2 Activity Assay Kit (SensoLyte 390) at a temperature of 27 °C. Samples were diluted 1:10 using the assay buffer provided with the ACE2 Activity Assay Kit. The assay was performed in 384-well plates. Plasma samples, assay buffer only (background), or 4-methyl-coumaryl-7-amide (0.16–5 μM) as a reference standard were added to an OptiPlate-384 F reaction plate at 10 μL/well. Then, ACE2 substrate solution (0.05 mM 4-methyl-coumaryl-7-amide/Dnp in assay buffer) was added at 10 μL/well. After the reaction, fluorescence intensity (Ex/Em = 330 nm/390 nm) was measured at 5-min intervals for 3 h using an EnSpire plate reader (PerkinElmer, Waltham, MA, USA). The fluorescence intensity of samples at each time point was calculated from the fluorescence intensity of the samples. The slope of each sample (RFU/min) was determined from a linear approximation of the plot during the period from 30 to 90 min after the start of the measurement.

### Statistical analysis

Data are expressed as mean ± standard error of the mean. Differences were analyzed as follows. Two-way analysis of variance, followed by Bonferroni post hoc analysis, was performed to determine differences over time between adenine and control groups (Figs. 1, 2), AA and vehicle groups (Fig. 3A, B) or adenine and olmesartan groups (Figs. 5, 6 and S1). Unpaired t-tests were used to determine differences between AA and vehicle mice (Figs. 3C–F, 4). P values < 0.05 were considered statistically significant.

## Supplementary Information

Supplementary Information.
