# Supplementary material for: Tissue-specific expression of the SARS-CoV-2 receptor, angiotensin-converting enzyme 2, in mouse models of chronic kidney disease
Source: Sci Rep. 2021 Aug 19;11:16843. doi: 10.1038/s41598-021-96294-8 (PMC8377123; doi:10.1038/s41598-021-96294-8)

Supplementary Figure

Figure S1

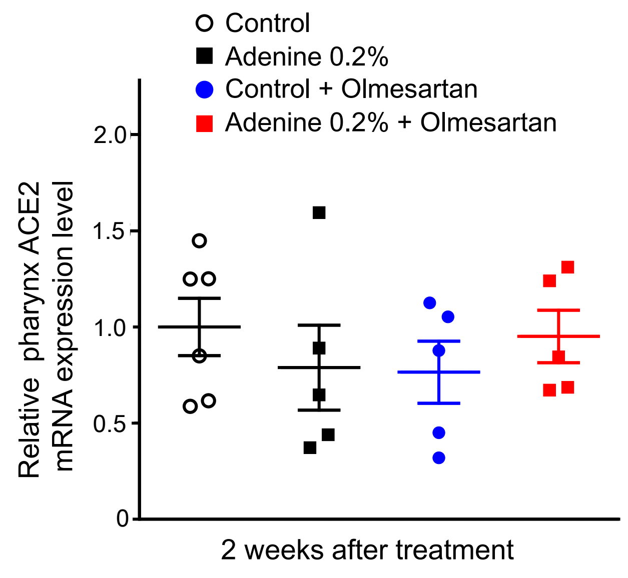

Supplementary Figure

Figure S2

(A)

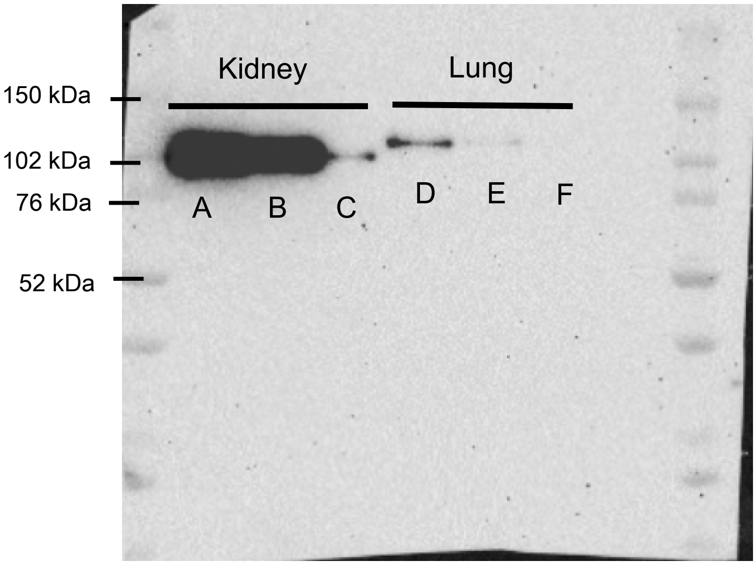

(B)

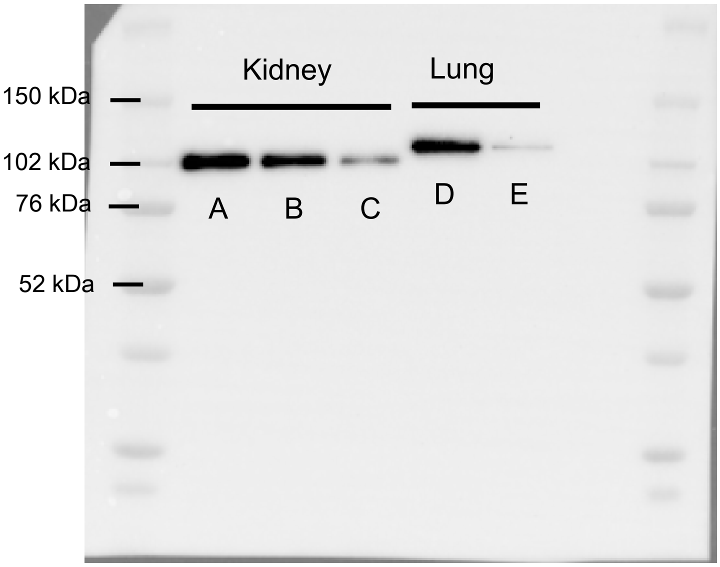

Supplementary Figure

Figure S3

(A) Kidney

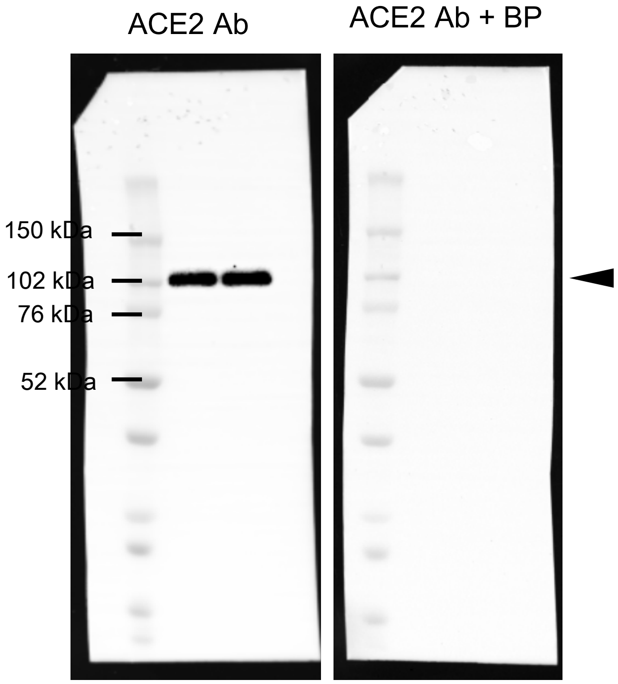

(B) Lung

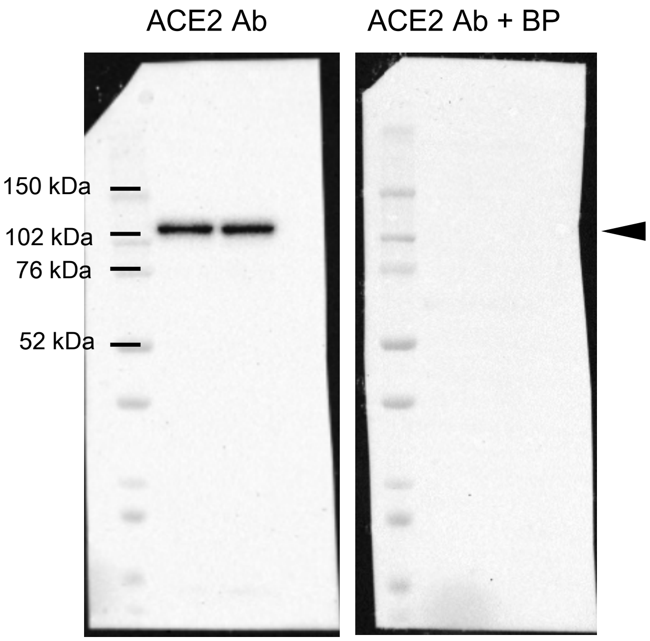

Supplementary Figure

Figure S4

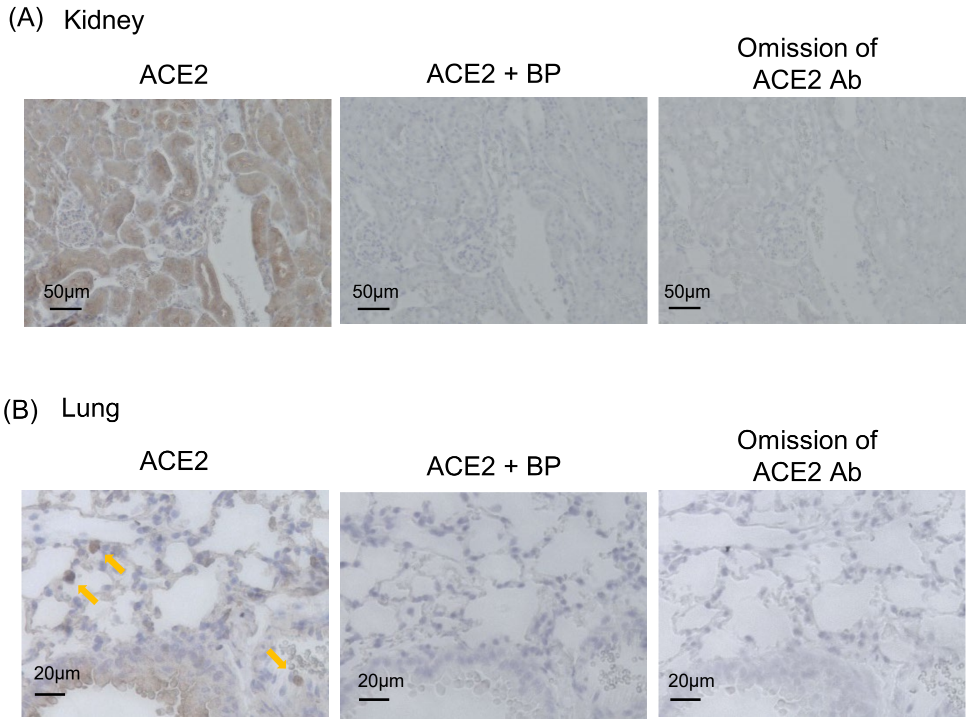

FIGURE LEGENDS

Supplementary Figure S1.

Effects of olmesartan administration on pharynx ACE2 mRNA expression levels.

Pharynx ACE2 mRNA expression levels in control, adenine, control-olmesartan, and adenine-olmesartan groups.

Supplementary Figure S2.

Western blot analysis on the same membrane for kidney and lung tissue.

(A) Pulmonary and renal ACE2 protein expression levels. 24 μg of total protein was applied to lane A, 10 μg of total protein was applied to lane B, 1.0 μg of total protein was applied to lane C, 24 μg of total protein was applied to lane D, 10 μg of total protein was applied to lane E and 1.0 μg of total protein was applied to lane F. Lane A, B, C; protein extracted from kidney tissue. Lane D, E, F; protein extracted from lung tissue.

(B) Pulmonary and renal ACE2 protein expression levels. 2 μg of total protein was applied to lane A, 1.5 μg of total protein was applied to lane B, 1.0 μg of total protein was applied to lane C, 24 μg of total protein was applied to lane D and 12

μg of total protein was applied to lane E. Lane A, B, C; protein extracted from kidney tissue. Lane D, E; protein extracted from lung tissue.

Supplementary Figure S3.

Western blot analysis of ACE2 protein in the kidney and lung.

The result of western blot analysis showed a single protein band of in tissue extracts derived from (A) kidney and (B) lung. This single band was not observed when the antibody was preabsorbed with an ACE2-selective blocking peptide. These results demonstrate the specificity of ACE2 antibody used in the present study. Ab, antibody; BP, blocking peptide.

Supplementary Figure S4.

Immunohistochemical analysis of ACE2 protein in the kidney and lung.

ACE2 staining in (A) kidney and (B)lung tissues was observed, which was not observed when the antibody was preabsorbed with an ACE2-selective blocking peptide or omission of ACE2 antibody. These results demonstrate the specificity of ACE2 antibody used in the present study. Ab, antibody; BP, blocking peptide.

Full unedited gel for Figure

Figure 2C

ACE2

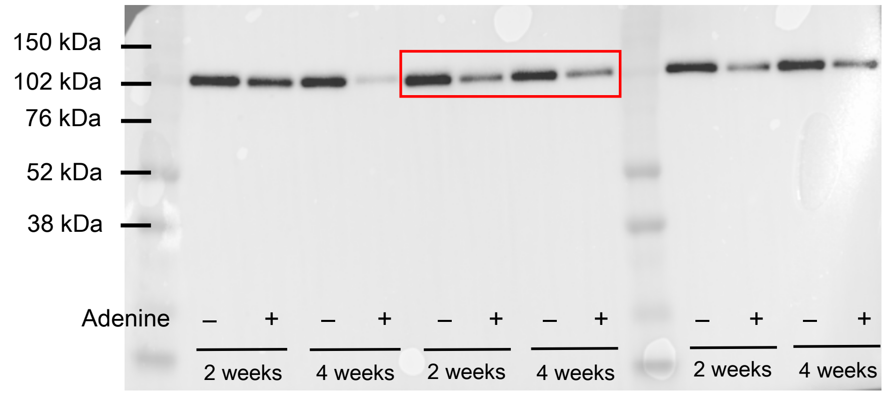

B-actin

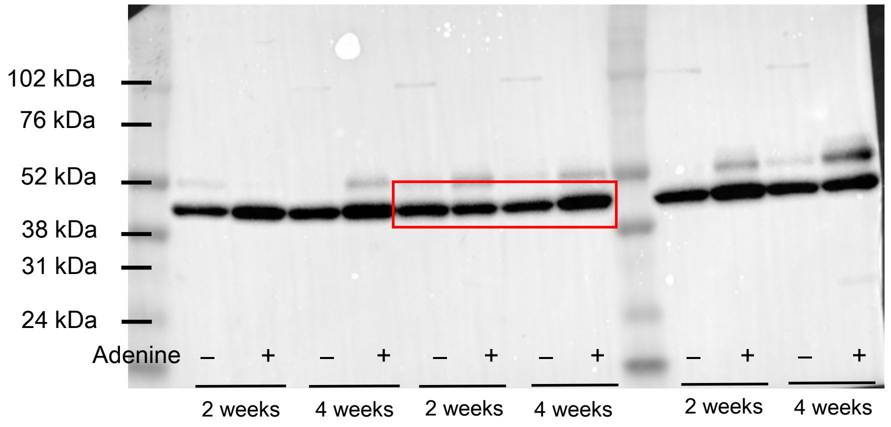

Figure 2D

ACE2

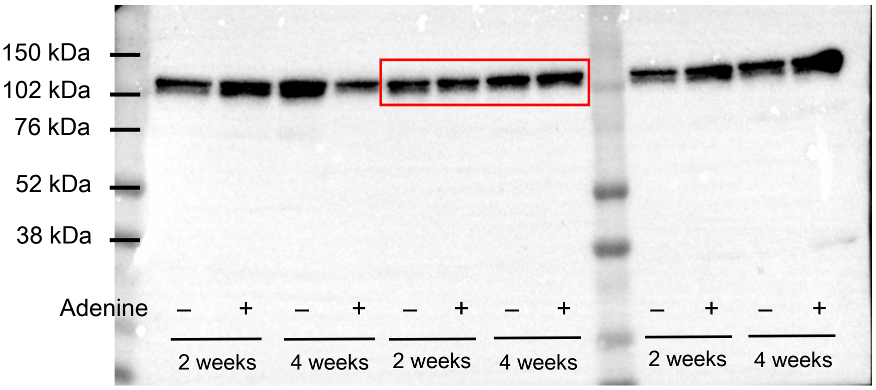

B-actin

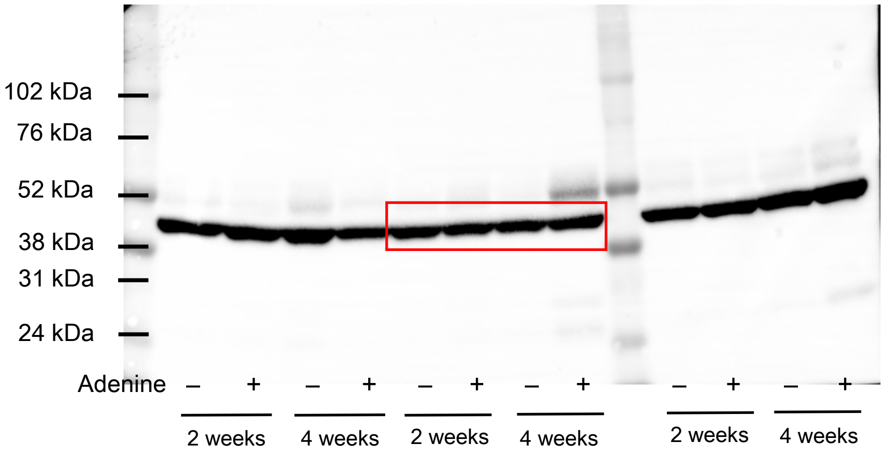

Figure 4C

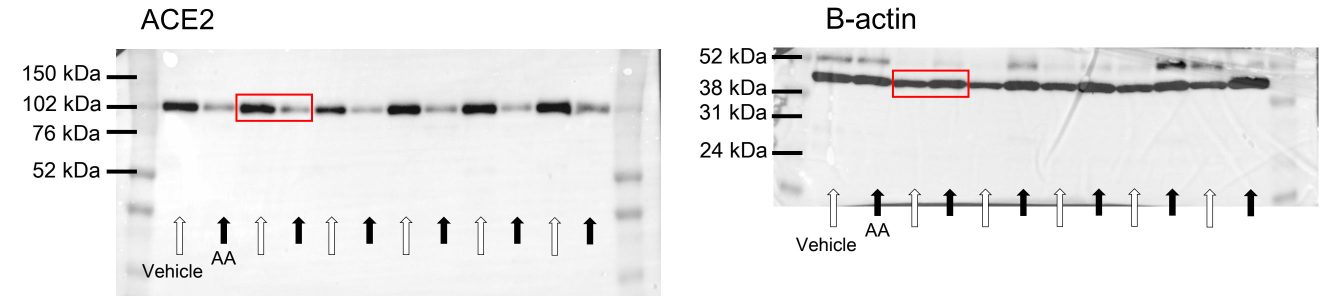

Figure 4D

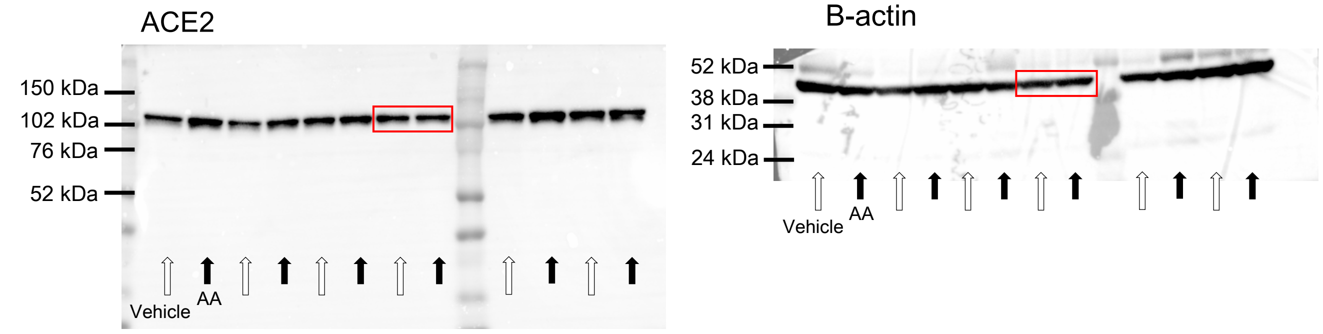

Figure 6A

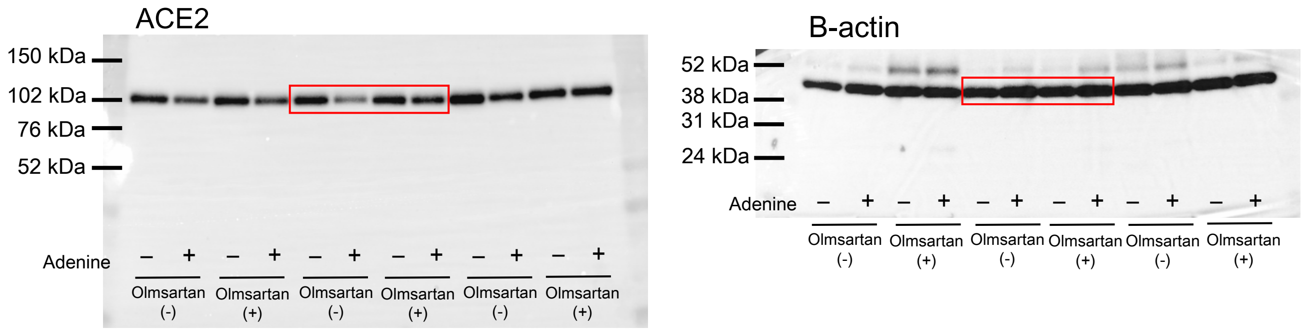

Figure 6B

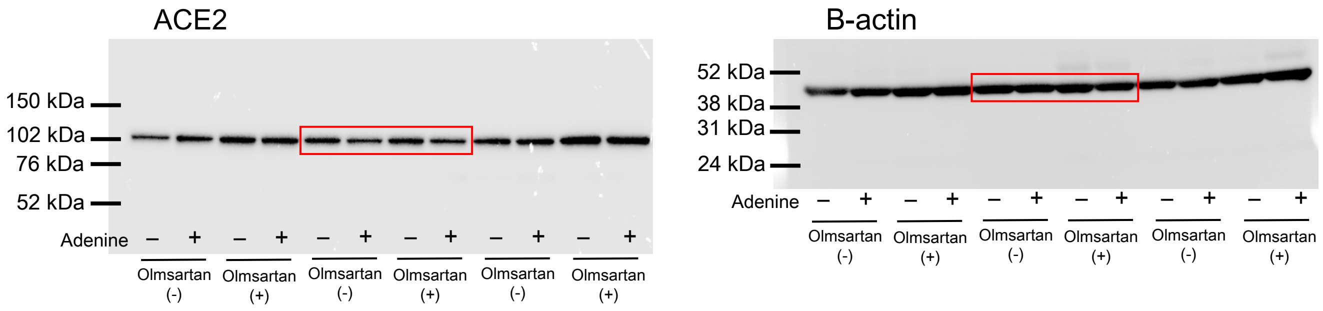

Supplement: Supplementary file 1 — Supplementary Information. [file 41598_2021_96294_MOESM1_ESM.pdf]
